# Comparison of Physical Performance and Muscle Thickness Between Older Women with High and Low Fall Risk: A Bayesian Approach

**DOI:** 10.3390/geriatrics11020044

**Published:** 2026-04-10

**Authors:** Claudineia Matos de Araujo, Rafael Pereira, Joanderson Felipe Soares Silva, Cláudia Thais Pereira Pinto, Alinne Alves Oliveira, Luciano Magno de Almeida Faria, Ludmila Schettino, Mikhail Santos Cerqueira, Marcos Henrique Fernandes

**Affiliations:** 1 Integrative Physiology Research Center, Department of Biological Sciences, Universidade Estadual do Sudoeste da Bahia (UESB), Jequié 45210-506, Bahia, Brazil; claudineia.matos@uesb.edu.br (C.M.d.A.); rafaelpereira@uesb.edu.br (R.P.); joandersonfelipess.contato@gmail.com (J.F.S.S.); cllaudia.thais@gmail.com (C.T.P.P.); alinnealvesoliveira@uesb.edu.br (A.A.O.); luciano.magno@uesb.edu.br (L.M.d.A.F.); lsrpaula@uesb.edu.br (L.S.); 2 Research Group in Neuromuscular Physiology, Department of Biological Sciences, Universidade Estadual do Sudoeste da Bahia (UESB), Jequié 45210-506, Bahia, Brazil; 3 Postgraduate Program in Nursing & Health, Universidade Estadual do Sudoeste da Bahia (UESB), Jequié 45210-506, Bahia, Brazil; marcos.fernandes@uesb.edu.br; 4 Center for Studies in the Epidemiology of Aging (NEPE), Universidade Estadual do Sudoeste da Bahia (UESB), Jequié 45210-506, Bahia, Brazil; 5 Postgraduate Program in Physical Education, Universidade Estadual do Sudoeste da Bahia (UESB), Jequié 45210-506, Bahia, Brazil

**Keywords:** aging, Bayesian analysis, body composition, functional mobility

## Abstract

**Objective:** The present study aimed to compare muscle thickness and physical performance in different functional tests predicting falls between older adults with low and high fall risk. **Methods:** Seventy-one community-dwelling older women (74.5 ± 8.5 years old) volunteered for this study. The Berg Balance Scale (BBS) was used to stratify the sample as low and high risk for fall (BBS cutoff = ≥ 50 points). The performance in the Timed Up and Go Test (TUGT), 5-repetition sit-to-stand test (5xSST), 3 m walk test (3mWT), and 3 m backward walk test (3mBWT) was assessed. The elbow flexor and knee extensor muscle thickness were obtained by ultrasound (USD). A linear mixed model analysis was used to determine between-group differences in functional mobility and muscle thickness, and Bayesian analysis was applied to check the probability to replicate the same results (i.e., the magnitude of the evidence). **Results:** The low-fall-risk group exhibited significantly better performance only in 3mWT (mean difference = 0.84 s [95% CI: 0.40 to 1.29 s]; *p* = 0.001) and 3mBWT (mean difference = 1.54 s [95% CI: 0.21 to 2.85 s]; *p* = 0.024). The Bayes Factor (BF) for performance on the 3mWT and 3mBWT shows that the low-fall-risk group has a probability of 98.7% (BF10 = 77.3) and 99.7% (BF10 = 368), respectively, of performing better than the high-fall-risk group. **Conclusions:** Based on inferential and Bayesian analysis, the performance in 3mWT and that in 3mBWT were classified as very strong to excellent instruments, respectively, for differentiating older women with high fall risk.

## 1. Introduction

Aging is associated with changes in body composition, such as progressive loss of lean mass and muscle strength, which influences anthropometric and functional parameters in older adults, being risk factors associated with increased falls [1]. Approximately one in three older adults fall each year, representing a common clinical problem in the elderly population, more prevalent in females, likely due to greater physical frailty, lower lean mass, and lower muscle strength [2,3].

Fall events in older adults result from postural instability, with balance impairment recognized as its main predictor [4]. In this sense, susceptibility to falls is associated with decline in functional performance, reduced effectiveness of postural responses, reduced sensory acuity, musculoskeletal and neuromuscular impairment, deconditioning associated with inactivity, and psychological and environmental factors [5]; this susceptibility has been evaluated through measures of muscle strength and mass, measures of autonomy and mobility/functionality, and instruments that assess fall risk. Thus, early identification of older women at higher risk of falls is an effective strategy for preventing possible negative outcomes, such as morbidity, mortality, and hospitalization due to falls [6,7].

Various instruments are used to assess fall risk, but the Berg Balance Scale (BBS) stands out as a qualitative measure consisting of functional clinical tests used to evaluate balance in older adults during daily activities [8,9]. The BBS is distinguished by its psychometric properties of reliability and validity, being considered the gold standard test for assessing fall risk, proving even more effective in predicting future falls than in confirming fall history in older adults [10,11,12,13].

Clinical tests developed to assess functional capacity are used to evaluate different dimensions of balance in older adults, to assist in clinical decisions regarding balance deficit and the development of fall prevention strategies. Studies evaluating factors associated with higher fall risk, as well as the relationship and complementarity of instruments that assess this risk, are increasingly necessary to help select ideal methods for fall-risk assessment, which can be a determining factor for screening and early intervention in older adults with high fall risk [14]. Performance in functional tests such as the Timed Up and Go Test (TUG), Five Times Sit-to-Stand Test (5xSST), and 6 m Walk Test (6mWT) are widely reported in the literature as factors associated with higher fall risk in older adults. The 3 m Backward Walk Test (3mBWT) is a recent proposal that also aims to assess fall risk in older adults. Initial results regarding association with falls in older women are promising, but studies comparing it with other fall predictors are still scarce.

Falls in older adults are a public health concern, and healthcare professionals’ ability to detect future falls through simple screening instruments is a fundamental element in their prevention and reducing risk factors, especially in individuals classified as high-fall-risk. Thus, this study aimed to compare performance in different functional tests predicting falls between older adults with low and high fall risk.

Previous studies [11,12,13] aimed to investigate the relationships of physical performance and anthropometric variables with fall risk, but we did not find studies comparing low- and high-risk older women, stratified based on BBS, using inferential and Bayesian methods, which is essential for future perspectives, since Bayesian analysis brings perspectives on the probability of replicating the same results. Thus, the present study aimed to compare muscle thickness and physical performance in various functional tests that predict falls between older adults with low and high fall risk. We used inferential and Bayesian methods to investigate this issue, and our main hypothesis is the presence of impaired muscle thickness and physical performance in high-fall-risk older women compared to low-fall-risk ones.

## 2. Materials and Methods

### 2.1. Sample

Seventy-one community-dwelling older women (74.5 ± 8.5 years old) volunteered for this study. We chose to include only older women because they are prone to falls and experience more fall-related fractures than older men [15]. Aged 60 years or older, demonstrating independent ambulation, and absence of limb amputations or skin lesions that affected their gait pattern were the inclusion criteria, while acute diseases or cognitive impairment [16,17] were the exclusion criteria.

The local Research Ethics Committee approved (protocol n. 2.783.516) all procedures according to the Declaration of Helsinki, and all volunteers signed the Informed Consent Form.

### 2.2. Proceedings

#### 2.2.1. Berg Balance Scale and Sample Stratification

For functional balance assessment, the Berg Balance Scale (BBS) was used to evaluate balance based on 14 functional daily living activities. These activities involve static and dynamic balance control tasks, which include sitting, standing, and leaning, among others, and indicate the subject’s balance when performing motor activities, with an overall score ranging from 0 to 56 (i.e., severely impaired to best performance/excellent balance) [8,9].

A meta-analysis conducted by Lusardi [18] identified a cut-off point of 50 points on the BBS for predicting future fall risk; therefore, this was adopted in this study as the cut-off point for the stratification of older women with high (≥50 points) or low (<50 points) fall risk.

#### 2.2.2. Physical Performance Evaluation

Functional mobility assessment was performed using the Timed Up and Go Test (TUGT), which is a sensitive and specific measure for identifying older adults at risk of falling and is widely used to assess functional mobility in this population [19]. The lower limb strength was assessed using the “5-repetition sit-to-stand test” (5xSST) as used by Pinheiro [20]. The 3 m walk test (3mWT) [21] and the 3 m backward walk test (3mBWT) [22] were used to assess gait performance.

#### 2.2.3. Muscle Thickness Measurements

Transverse ultrasound images were obtained from the right Brachial (Br), Biceps Brachial (BB), Vastus Lateralis (VL), and Rectus Femoris (RF) muscles using a B-mode 2-dimensional ultrasound (USD) imaging device (Figlabs^®^ FP 102, Saevo, Ribeirão Preto, São Paulo, Brazil), as described in Matos De Araujo [23]. A linear array transducer (Figlabs^®^, L471, sampling frequency of 7.0 MHz) was used, and a single trained and qualified evaluator performed all USD measurements, which were obtained with volunteers remaining upright, with their upper limbs relaxed and with palms facing forward; the transducer was positioned perpendicularly to muscular tissue and underlying bone as described by Takai [24].

The elbow flexor muscle thickness was recorded at 60% of the distance between the lateral epicondyle and the acromial process, as proposed by Abe [25]. During the image recording, the pressure was kept to a minimum to avoid excess compression and distortion, and a generous amount of contact water-soluble gel was applied [26].

Subcutaneous adipose tissue at the tissue–muscle and muscle–bone interfaces was identified in the USD image and used to determine the muscle limits, guiding the muscle thickness measurements for each of the elbow flexor (EF) and knee extensor (KE) muscle groups as performed by Matos De Araujo [23].

**Figure 1:**
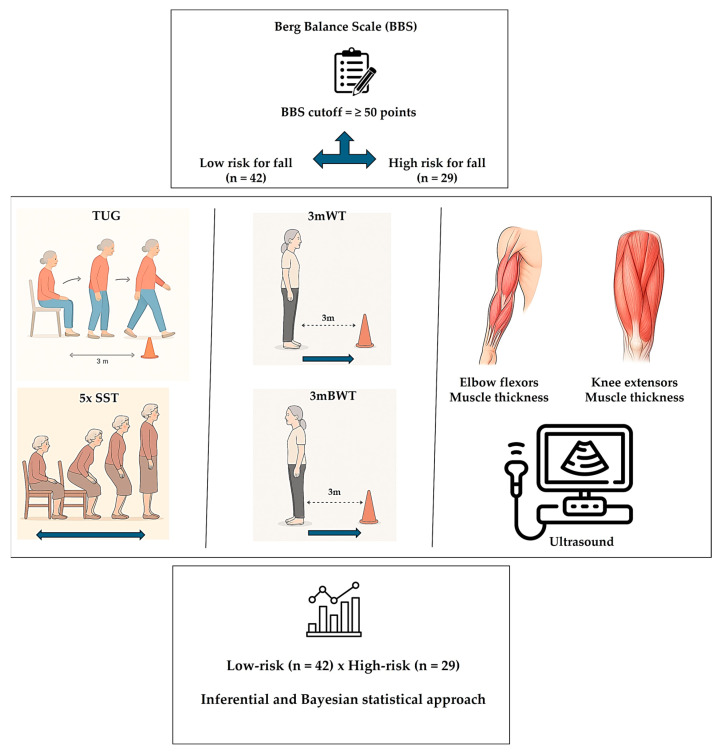
Experimental flow: Sample stratification based on Berg Balance Scale (low- x high-risk). Physical performance evaluation and muscle thickness measurement. Inferential and Bayesian procedures to test our hypothesis. Timed Up and Go Test (TUG); “5-repetition sit-to-stand test” (5xSST); 3 m walk test (3mWT); 3 m backward walk test (3mBWT).

Figure 1 presents the experimental flow applied in our study.

### 2.3. Statistical Analysis

A linear mixed model analysis was used to determine between-group differences in functional mobility and muscle thickness, taking groups as a fixed factor and age as a random factor. The normality of the data was not checked since the linear mixed model analysis used is reportedly robust in addressing Type 1 error rates when analyzing non-normal data [27]. The critical alpha was set at 0.05, and all procedures were carried out in SPSS version 21.0 (IBM Corp., Armonk, NY, USA).

Results are presented as mean ± SD, mean difference between groups, and 95% confidence interval (95% CI). The mean differences and their 95% CIs are reported and were interpreted as a measure of effect size, as this approach allows identifying the direction and magnitude of the effect, justifying its use as an adequate measure of effect size [28]. To check the probability of replicating the same results (i.e., the magnitude of the evidence), we applied the Bayes Factor (BF) hypothesis-testing analyses [29,30,31]. Individual comparisons were based on the default *t*-test with a Cauchy (0, r = 1/√2) prior. The outcomes were classified as anecdotal (BF10 = 1–3), moderate (3–10), strong (10–30), very strong (30–100), and extreme (>100) favoring the alternative hypothesis, or anecdotal (BF10 = 1–0.33), moderate (0.33–0.1), strong (0.1–0.03), very strong (0.03–0.01), and extreme (<0.01) favoring the null hypothesis (Lee and Wagenmakers’ classification) [32,33]. To calculate the probability of finding the same results again, we divided the actual BF_10_ value by BF_10_ + 1. All BF analyses were performed using Jamovi (Version 2.3.15; The Jamovi Project, Sydney, Australia) [34].

## 3. Results

### 3.1. Sample Characterization

The mean age of the studied older adults was 74.5 ± 8.5 years old. When stratified according to fall risk, the high-fall-risk group was significantly older than the low-fall-risk group (low-fall-risk group = 70.8 ± 6.2 years old; high-fall-risk group = 79.7 ± 8.6 years old; *p* = 0.0001). Weight (low-fall-risk group = 66.9 ± 9.4 kg; high-fall-risk group = 63.1 ± 8.6 kg; *p* = 0.141), height (low-fall-risk group = 152.9 ± 6.5 cm; high-fall-risk group = 151.8 ± 6.1 cm; *p* = 0.248), and body mass index (low-fall-risk group = 28.7 ± 4.1 cm; high-fall-risk group = 27.3 ± 4.8 cm; *p* = 0.301) were not significantly different between the high- and low-risk groups. Table 1 presents data from chronic diseases, such as diabetes and osteoarthritis, the history of falls in the last 12 months, and the usage of psychotropic medications from the studied older women according to the fall risk.

### 3.2. Inferential and Bayesian Statistics

Only the performance in the walking tests (i.e., forward and backward) demonstrated a significant between-group difference (*p* < 0.05). The low-fall-risk group exhibited significantly better performance (3mWT: mean difference = 0.84 s [95% CI: 0.40 to 1.29 s]; *p* = 0.001/3mBWT: mean difference = 1.54 s [95% CI: 0.21 to 2.85 s]; *p* = 0.024). The Bayes Factor analyses for performance on the 3mWT and 3mBWT show that the low-fall-risk group has a probability of 98.7% (BF10 = 77.3) and 99.7% (BF10 = 368), respectively, of performing better than the high-fall-risk group. The performance in the TUGT exhibited a moderate [85.7% (BF10 = 6.00)] probability of being better in the low-fall-risk group, while the elbow flexor muscle thickness exhibited an anecdotal [56.9% (BF10 = 1.32)] probability of being greater in the low-fall-risk group. In contrast to other variables, the 5xSST and knee extensor muscle thickness exhibited a posterior probability of 30.7% (BF10 = 0.444) and 47.9% (BF10 = 0.921) between groups, respectively, classified as anecdotal favoring the null hypothesis. The results from inferential and Bayesian statistics are presented in Table 2.

## 4. Discussion

This study aimed to compare performance in different functional tests predicting falls between older women with low and high fall risk. After age-adjusted comparison, we found that the performance in 3mWT and 3mBWT was statistically better in the low-fall-risk group. Bayesian analysis indicated a very strong (98.7%) extreme probability (99.7%) of a between-group difference, for 3mWT and 3mBWT, respectively, indicating that they are excellent parameters for differentiating older women with low and high fall risk.

Walking performance is recognized as a relevant parameter for measuring functionality in a wide variety of populations, being attested as a “vital sign” [35]. Indeed, the act of walking depends on a set of factors, such as strength, postural balance, spatial orientation, proprioception, and ability to make constant and rapid adjustments to position changes, among others, which makes the measures of gait performance an excellent instrument for discriminating people with low and high fall risk, especially older adults, since aging is associated with a natural decline in all factors that influence gait performance.

Indeed, walking without adequate visual feedback, as in backward walking, makes it difficult to plan foot support position, generating slower movement to maintain safety, which will tend to be even slower in older adults who tend to exhibit sensorimotor impairment [36]. In this context, walking backward may be a particularly important factor as a fall predictor in individuals with any condition that prevents this task [37], especially because few daily situations require the execution of this type of locomotion that primarily depends on good sensorimotor integration.

Based on the premise that the older adults present sensorimotor decline, impacting the ability to integrate sensory information and generate adequate motor responses, applying motor challenges, like walking backward, may highlight differences in motor competence between older adults who are less or more prone to falls, which perhaps cannot be detected with more conventional tests. Our results corroborate this premise, since in the present study, volunteers presented good motor capacity (mean BBS score = 49.5 points; median = 51 points), and tests widely reported as excellent fall predictors in older adults, such as TUGT and 5xSST, showed no difference between the studied groups. Additionally, muscle mass also does not seem to be a determinant of low- and high-fall-risk conditions in older women with the characteristics of our study sample.

Our results corroborate those of Fritz [38], indicating that backward gait performance seems to be the best predictor of fall risk in older adults. However, in that study, the sample exhibited a mean age 10 years higher than in our study sample. Additionally, the authors stratified the older adults according to fall history, while in our study, we decided to stratify according to a recognized cut-off point in BBS. This choice is based on the fact that BBS demonstrates a good ability to predict future falls, especially when considering high cut-off points (>45 points) [4,39].

This recognized property of BBS in predicting future falls, without necessarily being associated with fall history, may justify the absence of an association between a low BBS score (cut-off at 50 points) and fall history observed in our study (*p* = 0.160). The fact that we adopted a higher cut-off point, following the findings from Lusardi [18], may have influenced this result.

In summary, our results support the hypothesis that gait performance, forward, but especially backward, may be more sensitive in identifying age-related factors, such as mobility and balance, being a promising clinical tool for assessing fall risk in older women [38,40].

It is worth emphasizing some methodological aspects and possible limitations of this study. Regarding the limitations, the cross-sectional design does not permit any conclusions to be drawn about individual changes in the studied variables over time. Additionally, the sample was limited to older women, predominantly those with good functional mobility, as indicated by a mean score of 49.5 on the BBS (median = 51 points). However, these two issues could point to a relevant aspect of this study, since women are more prone to falls, and among subjects with higher functional mobility, as studied herein, it will be harder to stratify older adults according to the propensity to fall.

## 5. Conclusions

The results of this study verified that gait performance, walking forward (3mWT), but also especially backward (3mBWT), has great potential to differentiate older women more prone to falls. Additionally, based on Bayesian analysis, the performance in 3mWT and that in 3mBWT were classified as very strong to excellent instruments, respectively, for differentiating older women with high fall risk, and TUGT, despite the absence of statistical differences in inferential statistics, also showed potential for this purpose, but with a moderate probability of differentiating. The good functional mobility of the studied sample could have conditioned these results, highlighting the need to implement motor challenges, such as walking backward, to stratify active older women with low and high fall risk.

## Figures and Tables

**Table 1:** Sample characteristics. Data are reported as absolute and relative frequencies [n (%)].

Variable		Low Risk (n = 42)	High Risk (n = 29)	*p* Value
Diabetes	No	33 (78.6)	24 (82.8)	0.663
	Yes	9 (21.4)	5 (17.2)	
Osteoarthritis	No	33 (78.6)	21 (72.4)	0.550
	Yes	9 (21.4)	8 (27.6)	
Fall in the Last 12 Months	No	31 (75.6)	18 (60.0)	0.160
	Yes	10 (24.4)	12 (40.0)	
Psychotropic Medications *	No	41 (97.6)	25 (86.2)	0.151
	Yes	1 (2.4)	4 (13.8)	

(*) antihistamines, antidepressants, benzodiazepines.

**Table 2:** Inferential and Bayesian analysis for the comparison of studied variables between community-dwelling senior women stratified according to the Berg Scale Score (cut off ≥ 50 points).

	Groups *		Mean Difference (95% CI)	*p* Value	BF_10,*U*_	Probability %
	Low-Risk (*n* = 42)	High-Risk (n = 29)				
TUGT (s)	8.98 (1.94)	10.63 (3.02)	0.53 (−0.71 to 1.77)	0.398	6.00 ^M^	85.7
5xSST (s)	12.21 (3.00)	13.18 (3.93)	0.78 (−1.05 to 2.60)	0.389	0.444 ^a^	30.7
3mWT (s)	3.34 (0.66)	4.19 (1.19)	0.84 (0.40 to 1.29)	0.001	77.3 ^V^	98.7
3mBWT (s)	5.16 (1.46)	7.96 (3.70)	1.54 (0.21 to 2.85)	0.024 *	368 ^E^	99.7
EF_MT (cm)	2.64 (0.43)	2.40 (0.47)	−0.13 (−0.41 to 0.14)	0.323	1.32 ^A^	56.9
KE_MT (cm)	3.45 (0.66)	3.16 (0.54)	−0.10 (−0.47 to 0.26)	0.576	0.921 ^a^	47.9

Timed Up and Go Test (TUGT); Five-repetition Sit-to-Stand Test (5×SST); 3 m Walk Test (3mWT); 3 m Backward Walk Test (3mBWT); Elbow Flexor Muscle Thickness (EF_MT); Knee Extensor Muscle Thickness (KEMT); Bayes Factor (BF_10_). (*) Data presented as mean (standard deviation). Superscript letters denote the strength of the Bayesian evidence: ^a^ = anecdotal; ^M^ = moderate; ^V^ = very strong; ^E^ = extreme; ^A^ = anecdotal favoring the null hypothesis.

## Data Availability

The data supporting the findings of this study are not publicly available due to privacy and ethical restrictions.
